# When and how to use ambulatory blood pressure monitoring and home blood pressure monitoring for managing hypertension

**DOI:** 10.1186/s40885-024-00265-w

**Published:** 2024-04-01

**Authors:** Eun Mi Lee

**Affiliations:** Division of Cardiology, Department of Internal Medicine, Wonkwang University Sanbon Hospital, Gunpo, Gyeonggi-do 15865 Republic of Korea

**Keywords:** Hypertension, Blood pressure, Ambulatory blood pressure monitoring, Home blood pressure monitoring

## Abstract

Many individuals have different blood pressure (BP) values in the office setting compared to that outside the office setting. Therefore, confirming hypertension based on office BP (OBP) measurement alone can lead to misdiagnosis and mistreatment. The limitations of OBP measurement have led to the complementary use of out-of-office BP measurements, including 24-hour ambulatory blood pressure monitoring (ABPM) and home blood pressure monitoring (HBPM). This review aims to describe when and how ABPM or HBPM can be used to accurately diagnose and treat hypertension. Both methods should be performed using validated automated oscillometric devices. To minimize user errors, ABPM should be performed using standard techniques, whereas HBPM requires patient education regarding proper BP measurements. ABPM provides short-term comprehensive information on BP, including daytime, nighttime, morning, and 24-h BP. Therefore, ABPM is recommended for the initial diagnosis of hypertension, assessment of BP phenotypes and circadian patterns, and detection of nocturnal hypertension, Furthermore, ABPM plays a critical role in confirming true resistant hypertension thereby excluding pseudo-resistant hypertension. However, it is not suitable for long-term follow-up of patients with hypertension. In contrast, HBPM involves multiple BP readings taken at specific times during the day and evening over a long period. Therefore, HBPM is recommended for diagnosing hypertension and assessing BP phenotypes. However, this method has limitations in measuring nocturnal BP and circadian BP patterns. HBPM is preferred over ABPM for the long-term follow-up of patients with hypertension. This approach improves patient adherence to treatment and ultimately enhances the rate of control of hypertension. Additionally, both methods play an important role in diagnosing and treating white coat hypertension during pregnancy. Consequently, out-of-office BP measurement is essential to prevent the misdiagnosis and mistreatment of hypertension. However, these two methods offer different information regarding the BP status of an individual, and they indeed show discrepancies in the diagnosis of hypertensive phenotypes. Therefore, it is crucial to understand the advantages and limitations of both ABPM and HBPM to ensure their appropriate use in clinical practice.

**Graphical Abstract:**

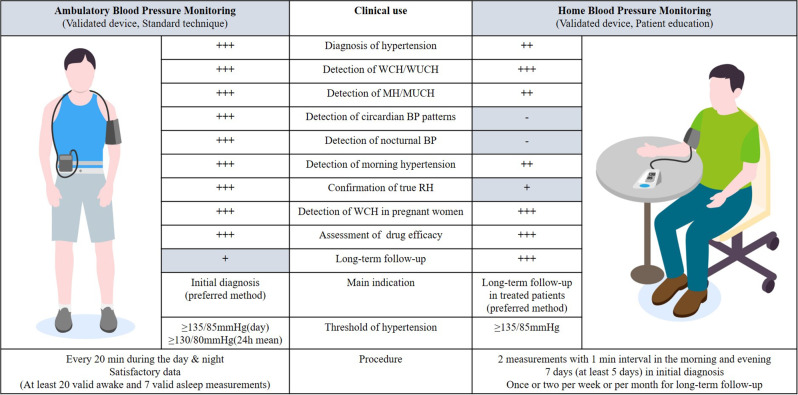

## Background

Hypertension is the primary cause of cardiovascular disease in Korea, [1] and accurate diagnosis and treatment of hypertension are critical for reducing cardiovascular morbidity and mortality. However, blood pressure (BP) fluctuates inherently, and many individuals have different BP readings in office settings compared to those outside the office. Thus, confirming hypertension based on office BP (OBP) measurement alone may lead to misdiagnosis and mistreatment. These limitations of OBP measurements have led to the complementary use of out-of-office BP measurements [2–4]. The major advantage of this method is its ability to provide multiple BP readings, away from medical settings, during routine daily activities. In addition, it is a stronger predictor of cardiovascular disease risk than OBP [5–10]. Out-of-office BP measurement comprises two techniques: 24-h ambulatory blood pressure monitoring (ABPM) and home blood pressure monitoring (HBPM). Among these, ABPM assesses both daytime and nighttime BP readings over 24 h, providing short-term comprehensive information on BP. However, it is inappropriate for long-term follow-up of patients with hypertension [11, 12]. Conversely, HBPM provides multiple BP readings at specific times during the day and night over a prolonged period, leading to improved patient adherence and physician inertia, and ultimately increased BP control rate [13, 14]. However, it is not suitable for detecting nocturnal BP values and all users require education on proper BP measurement [12, 15, 16]. Thus, these two methods offer somewhat different information on the BP status of an individual for the diagnosis and treatment of hypertension.

Therefore, we aimed to describe when and how ABPM or HBPM should be used for the proper diagnosis and treatment of hypertension in clinical practice.

## Ambulatory blood pressure monitoring

### Advantages and limitations

ABPM provides comprehensive information on BP, including daytime, nighttime, morning, and 24-h BP, thereby enabling the assessment of circadian BP patterns, BP phenotypes, and short-term BP variability.

Therefore, ABPM is preferred over HBPM for the initial diagnosis of hypertension; however, it is not suitable for long-term follow-up [4]. Yacong et al. reported that ABPM had excellent reproducibility at the population level, whereas the reproducibility of intra-individual BP values and dipping status from a 24-h ABPM was limited [17]. Poor intra-individual reproducibility may be related to differences in daily activities, decreased accuracy at higher BP, quality of sleep, and probably reduced accuracy of the device under real ambulant conditions [18]. Moreover, conventional ABPM is not widely feasible in primary clinics, is not acceptable for frequent use, and may cause problems such as sleep disturbance, anxiety, and skin irritation during monitoring owing to cuff inflation [19]. To overcome these shortcomings, cuffless BP monitoring using a wearable device has been attempted for continuous BP estimation [20]. However, currently, cuffless devices are not recommended for use in the diagnosis or management of hypertension in clinical practice [4]. HBPM may be considered for patients unwilling to undergo ABPM or for those who previously experienced considerable discomfort during ABPM [4]. 

### Device selection and procedures for ABPM (Fig. 1)

**Fig. 1:**
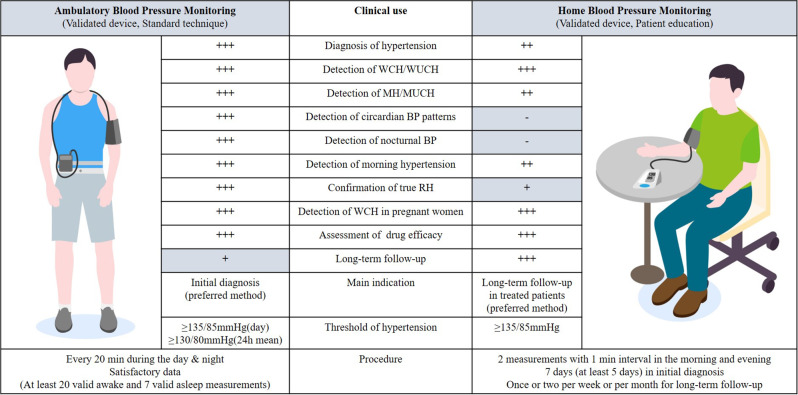
Clinical use of ABPM and HBPM. This figure shows the standard protocols and clinical indications for ABPM (left) and HBPM (right). The correct posture for ABPM is illustrated on the right. A small digital blood pressure monitor was attached to a belt around the patient’s waist and connected to a cuff around the upper arm. The cuff is wrapped around the non-dominant upper arm (usually left) at the heart level. It is to remain still with the arm relaxed during each measurement. The standard posture of HBPM is illustrated on the left: sitting in a chair with the back straight and supported, legs uncrossed, feet kept on the floor, bare arm rested on the table, cuff to fit arm circumference, and cuff placed mid-arm at heart level. The indications for ABPM and HBPM are listed in the center. The degree of recommendation is indicated by the degree of the plus mark: +++, strong recommendation; ++, moderate recommendation; +, weak recommendation; -, no recommendation, owing to the limited role of HBPM. ABPM, ambulatory blood pressure monitoring; HBPM, home blood pressure monitoring; WCH, white coat hypertension; WUCH, white coat uncontrolled hypertension; MH, masked hypertension; MUCH, masked uncontrolled hypertension; BP, blood pressure; RH, resistant hypertension; 24 h, 24 h; min, minutes

It is important to use a cuff-based, validated, upper arm oscillometric device according to established protocol. Separate validation is required in specific populations, such as children and pregnant women, for conventional 24-h ABPM [21]. Up-to-date lists of validated devices are available on both global and regional websites (www.stridebp.org, www.medaval.ie, www.dableducational.org, and www.validatebp.org) [22]. ABPM is programmed to take BP readings every 15–30 min during the day and every 30–60 min at night. It was performed on the non-dominant arm with an appropriate cuff according to the device instructions [11, 12]. The recently published 2023 ESH (European Society of Hypertension) Guidelines advise measuring blood pressure every 20 min throughout the day and night to minimize the risk of missing measurements and ensure the reliability of ABPM [4]. It is necessary to provide instructions for ABPM to patients, to preferably perform with usual daily activities and nighttime sleep, to remain still with the arm relaxed during each measurement, and to maintain a diary. ABPM data were considered satisfactory with 24-h recording with at least 70% of expected measurements, at least 20 valid awake measurements, and seven valid asleep measurements [11, 12]. 

## Home blood pressure monitoring

### Advantages and limitations

HBPM assesses multiple BP readings outside the office setting in the usual environment of each individual. In addition, it is easy and relatively inexpensive to use. Thus, it can be used over a prolonged period, and its data are more reproducible than those obtained from office BP measurements [12, 15, 16]. Consequently, it provides insight into BP phenotypes such as white coat hypertension (WCH) and masked hypertension (MH), and long-term variability in BP [12, 15, 16]. Furthermore, it allows long-term follow-up of treated hypertension. However, HBPM is generally not well suited for assessing nocturnal hypertension and nocturnal BP dipping status, and may induce anxiety in some patients [12, 15, 16]. Additionally, since it is performed by the patients themselves without medical supervision, it is crucial to educate the patients regarding the standardized protocol to obtain accurate BP readings [3, 12, 15, 16]. 

### Device selection and procedures for HBPM (Fig. 1)

To reduce device errors, validated upper arm devices are generally recommended for HBPM because BP values are computed by predetermined algorithms that are known only to the manufacturer [3, 12].. Picone et al. reported that a few devices for HBPM globally have evidence of validation for accuracy [23]. Moreover, devices may be inaccurate in arrhythmias, pregnancy, and pediatrics, [16] but recently, devices that can detect arrhythmias, such as atrial fibrillation, have become available on the market [24]. The standard protocol for HBPM is as follows: rest for 5 min before measurement, avoid smoking, alcohol, or caffeine for 30 min, and refrain from talking during and between measurements. For measurement, select a validated automated upper arm device, use the proper cuff size according to the device instructions, place the cuff at heart level, and take two measurements with 1-min interval in the morning and in the evening. The measurement should be performed for 7 days (at least 5 days) for the initial diagnosis, and duplicate measurements should be taken once or twice per week or per month for long-term follow-up. After measurement, all the readings should be accurately recorded in a BP log [3, 4, 12, 16]. The most common user errors for HBPM may include using the wrong-sized cuff, incorrect patient positioning, incorrect cuff placement, and using non-validated devices [25]. Therefore, ensuring that HBPM is measured correctly before using it in general practice is crucial.

### Clinical implications for using ABPM and HBPM (Fig. 1)

#### Diagnosis of hypertension

Hodgkinson et al. reported that an OBP > 140/90 mmHg showed a mean sensitivity and specificity of 74.6% for diagnosing hypertension compared to ABPM at 135/85 mmHg. In contrast, HBPM > 135/85 mmHg exhibited a mean sensitivity of 85.7% and specificity of 62.4% [26]. These findings suggest that neither OBP nor HBPM have sufficient sensitivity or specificity to be recommended as a single diagnostic test for hypertension [26]. Therefore, when OBP is elevated, guidelines recommend repeated measurements of OBP at subsequent office visits or out-of-office BP measurements using either ABPM or HBPM to confirm hypertension [2–4]. Ideally, both ABPM and HBPM should be employed to confirm hypertension as they provide somewhat different and complementary information regarding the BP status.

#### Diagnosis and treatment of WCH and WUCH

**Fig. 2:**
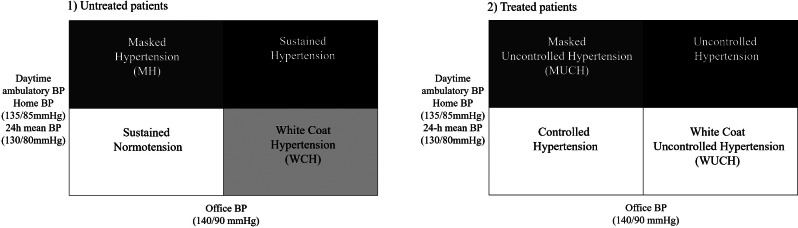
Classification of BP phenotypes and their cardiovascular risk in untreated and treated patients with hypertension. According to the results of OBP and out-of-office measurements, an individual’s BP status is categorized into four phenotypes in untreated and treated patients; normotension (both OBP and out-of-office BP not elevated), WCH (elevated OBP but not out-of-office BP, MH (elevated out-of-office BP but not OBP), sustained hypertension (elevated OBP and out-of-office BP) in untreated patients; controlled hypertension (controlled OBP and out-of-office BP), WUCH (elevated OBP but not out-of-office BP), MUCH (elevated out-of-office BP but not OBP), and uncontrolled hypertension (elevated OBP and out-of-office BP) in treated patients with hypertension. Black and dark gray colors indicate increased risk of cardiovascular disease; light gray show intermediate risk between normotension and sustained hypertension; white color indicates no increased cardiovascular risk. BP, blood pressure; OBP, office BP; WCH, white coat hypertension; WUCH, white coat uncontrolled hypertension; MH, masked hypertension; MUCH, masked uncontrolled hypertension; 24-h, 24-hour

Based on the results of the OBP and out-of-office measurements, the BP status was categorized into four phenotypes in untreated and treated patients [3, 4] (Fig. 2). The Korean Ambulatory BP Monitoring (Kor-ABP) Registry study showed that the discordancy rate between ambulatory BP and OBP in untreated and treated patients was 32.5% and 26.5%, respectively; WCH (14.9%) and MH (17.6%) in untreated patients; white coat uncontrolled hypertension (WUCH, 13.5%) and masked uncontrolled hypertension (MUCH, 13.0%) in treated patients. Therefore, approximately one-third of the patients with hypertension would have been misdiagnosed if they had considered OBP alone [27]. Kang et al. reported that HBPM has high specificity (86–94%) but low sensitivity (47–74%) compared to ABPM in the diagnosis of WCH and MH, regardless of treatment status [28]. This suggests that HBPM may complement ABPM in the diagnosis of WCH and WUCH. The ESH recommends that ABPM and/or HBPM be performed when WCH is suspected in patients with grade 1 hypertension [4]. Misdiagnosis can lead to over or undertreatment in real-world practice. Therefore, when BP is discordant between OBP and out-of-office BP, antihypertensive medications should be considered based on out-of-office BP. Compared to normotension, WCH was not an innocent condition [29–33]. Mancia et al. reported that WCH alone without hypertension-mediated organ damage (HMOD) is accompanied by a marked increase in new hypertension, new HMOD, and long-term risk of mortality compared to normotension [33]. Therefore, although patients with WCH should not initiate antihypertensive medication at this time, they should be recommended periodic BP monitoring with repeated ABPM or long-term HBPM and follow-up assessment of cardiovascular risk factors and HMOD [3, 4]. Lifestyle interventions are recommended for patients with WCH to reduce cardiovascular risk. Moreover, whether drug treatment should be used remains unresolved; however, it can be considered in patients with WCH, HMOD, and a high cardiovascular risk [4]. In contrast, among treated patients, those with WUCH did not have an increased cardiovascular risk compared to those with controlled OBP and controlled out-of-office BP [3, 4, 31]. Therefore, if OBP is elevated, ABPM or HBPM may be considered to rule out WUCH to avoid mistreating it as uncontrolled hypertension before up-titrating the antihypertensive medication. Additionally, repeat out-of-office BP measurements are recommended to confirm WCH and to detect the transition to sustained hypertension in individuals with WCH [3, 4]. 

#### Diagnosis and treatment of MH and MUCH

MH is associated with a high risk of progression to sustained hypertension, metabolic abnormalities, HMOD, and adverse clinical outcomes [29, 34–38]. The prevalence of MH also varies depending on the time of BP monitoring. However, who should be screened and how the screening should be performed for MH has not yet been established. Booth et al. reported that performing ABPM in all adults in the U.S. with non-elevated OBP who were not taking antihypertensive medications is impractical because of the low probability of detecting MH [39]. An OBP in the prehypertensive range (120–139/80–89 mmHg) is associated with a higher prevalence of MH. However, Viera et al. demonstrated that an OBP cutoff of 120/82 mmHg would result in a high false-positive rate of approximately 40% [40]. Thus, OBP alone may not be sufficient to detect MH. Several factors, including young age, male sex, cigarette smoking, alcohol drinking, anxiety, job stress, higher levels of physical activity, diabetes, chronic kidney disease, obstructive sleep apnea, exaggerated BP response to exercise, and orthostatic posture, might be predictors of MH [3, 4, 41]. Thus, individuals with normal or high-normal OBP accompanying multiple risk factors for MH or HMOD may be considered for screening for MH [3, 4, 35]. A previous systemic review and meta-analysis reported that the reproducibility of MH was better with ABPM than with HBPM (Kappa reliability test: 0.41 in ABPM and 0.26 in HBPM) [42]. Kang et al. demonstrated that HBPM has high specificity (86–94%) but low sensitivity (47.74%) in the diagnosis of MH [28]. Thus, HBPM may serve as a complementary method to, but not a replacement for, ABPM. Cohen et al. demonstrated that the Kappa statistics between the first and second ABPM for the detection of MH were 0.50 for masked awake, 0.57 for masked 24-h, 0.57 for masked asleep, and 0.58 for any MH. Thus, the authors suggest that clinicians should consider the moderate reproducibility of MH when interpreting the results of a single ABPM [43]. Accordingly, the diagnosis of MH requires confirmation with at least a second set of office and out-of-office BP measurements. No randomized clinical trials have evaluated the efficacy of drug treatments for MH, and the effects of drug therapy are therefore unknown. However, considering MH as a poor prognostic factor, the ESH guidelines recommend stringent lifestyle modifications and close monitoring of patients with confirmed MH [4]. Antihypertensive drug therapy may be considered for patients with MH who have a higher cardiovascular risk and HMOD [4]. In treated patients, MUCH is associated with a worse metabolic profile, HMOD, and adverse clinical outcomes [37, 38, 44]. However, an optimal strategy for screening and treatment of MUCH has not been established. In the Korean-ABP registry, high-normal office BP, underuse of antihypertensive drugs, dyslipidemia, prior stroke, and left ventricular hypertrophy were suggested as predictors of MUCH [45]. ESH guidelines recommend that up-titration of medication should be advised in patients with MUCH above the recommended target BP values if it is well tolerated [4]. However, there is no established evidence to evaluate the efficacy of drug treatment and the optimal BP target for MUCH [4]. Therefore, physicians should consider excluding MH or MUCH with at least repeated ABPM or HBPM in patients with multiple risk factors for MH, MUCH, or HMOD despite normal or high-normal office BP to prevent the undertreatment of hypertension.

#### Detection of circadian BP patterns, nocturnal hypertension, and morning hypertension

The quantification of circadian BP changes is a unique advantage of ABPM, leading to the identification of dipping patterns. However, this is the main limitation of HBPM. Nondipping and reverse dipping were associated with an increased risk of cardiovascular events [46, 47]. Nocturnal hypertension is defined as an average BP ≥ 120/70 mmHg recorded during the night hours with ABPM [4]. HBPM may have limitations in measuring nocturnal BP, although some home devices have shown a level of accuracy comparable to that of ABPM [48]. However, further research on the appropriate measurement protocol and clinical significance of HBPM for detecting nocturnal BP is needed. Nocturnal hypertension is closely related to cardiovascular events and HMOD in the general population and patients with hypertension [49–51]. However, nocturnal hypertension is poorly reproducible in both untreated and treated patients. In addition, nighttime dipping and nondipping phenotypes can shift from one phenotype to another during treatment [52]. Therefore, these findings suggest that assessing nocturnal hypertension with ABPM is useful for predicting increased cardiovascular risk, but quantification of nighttime phenotype should be based on repeated ABPM rather than only a single recording. Morning hypertension was defined as elevated ABPM or HBPM in the morning (≥ 135/85 mm Hg) and a normal clinical BP (< 140/90 mmHg) [53]. Guo et al. reported that HBPM might be preferred over ABPM for detecting morning hypertension because of its better reproducibility and stronger correlation with vascular indices [54]. MH and MUCH comprise morning, daytime, and nocturnal hypertension [55]. Therefore, physicians should consider excluding MH or MUCH due to uncontrolled nocturnal and morning hypertension if they pose an increased risk of cardiovascular disease, even if their daytime BP is well controlled [55]. 

The optimal treatment for nocturnal and morning hypertension has not yet been established; however, several therapeutic strategies such as bedtime dosing and salt restriction have been proposed [56]. In certain instances of uncontrolled nocturnal and morning hypertension, particularly among individuals with conditions such as diabetes mellitus, chronic kidney disease, and obstructive sleep apnea, bedtime dosing has been shown to be effective in lowering evening and early morning BP [56]. However, the Treatment in Morning versus Evening (TIME) study failed to demonstrate the benefit of bedtime dosing in reducing cardiovascular outcomes in patients with hypertension [57]. In addition, evening dosing is associated with poor drug compliance. Therefore, ABPM may be the preferred method, whereas HBPM may have a limited role in evaluating circadian BP patterns and nocturnal hypertension. Morning BP can be assessed using both ABPM and HBPM [53]. Currently, it is unclear whether routine bedtime dosing is beneficial for reducing cardiovascular outcomes [56]. 

#### Confirmation of true RH

Resistant hypertension (RH) is defined as hypertension uncontrollable by ≥ 3 antihypertensive drugs, and it is associated with higher mortality and morbidity than non-RH [58, 59]. Therefore, it is important to accurately identify RH for proper management. In the Spanish ABPM Registry, among 8,295 patients diagnosed with RH based on OBP, 37.5% had normal ambulatory BP (WUCH) [60]. Thus, WUCH has been misdiagnosed as having RH, which is known as pseudo-RH. Therefore, excluding WUCH by using ABPM is a critical step in confirming the true RH. However, HBPM may have limitations in measuring nocturnal BP, although nondipping and nocturnal hypertension are commonly observed in RH. Therefore, HBPM may play a complementary role, but cannot replace ABPM in the diagnosis of RH. However, HBPM is useful in improving the long-term management of RH [59, 61]. It may provide information on the response to antihypertensive medication and improve drug adherence, ultimately leading to an improved hypertension control rate and decreased medical costs.

#### Detection of WCH with proper devices in pregnant women

The number of cases of pregnancy-induced hypertension is increasing in Korea [62]. During pregnancy, BP directly affects the mother’s health and fetal development. Thus, accurate BP measurement during pregnancy is crucial for the diagnosis and management of hypertension. Pregnancy induces significant hemodynamic changes including increased blood volume, stroke volume, and heart rate [63]. These alterations lead to an increased cardiac output and decreased peripheral vascular resistance. In a systematic review, Bello et al. found that the majority of validation studies evaluating BP measurement devices during pregnancy had violations [64]. Therefore, guidelines recommend the use of specially validated BP devices for OBP and out-of-office BP measurements during pregnancy [4]. Currently, OBP remains the primary method for diagnosing and treating hypertension in pregnant women. WCH is a common phenomenon in pregnant women; therefore, proper use of out-of-office BP monitoring is necessary to rule out WCH [65]. Moreover, women with WCH before 20 weeks are associated with worse perinatal and maternal outcomes than normotensive women but have better outcomes than those with gestational hypertension and chronic hypertension [66]. Therefore, if OBP is elevated in untreated pregnant women, it may be useful to confirm the diagnosis of hypertension using ABPM or HBPM to avoid unnecessary treatment [67, 68]. Consequently, women with WCH require continued HBPM throughout pregnancy to assess the risk of developing preeclampsia, having a small-for-gestational-age baby, and experiencing preterm birth. In treated pregnant women, WUCH may be interpreted as uncontrolled hypertension or a hypertensive emergency, potentially leading to overtreatment or unnecessary interruption of pregnancy [68]. Therefore, ABPM or HBPM is required for the proper management of all pregnant women, regardless of gestational age.

#### Assessing the efficacy of antihypertensive treatment and long-term control of hypertension

In a systemic review and meta-analysis, Agarwal et al. demonstrated that HBPM-based therapy resulted in reduction of BP (mean systolic/diastolic BP changes, -2.63 mmHg/-1.68 mmHg), and these reductions were greater when telemonitoring was used. Further, it led to more reductions in antihypertensive medication use and was associated with less therapeutic inertia [13]. Tucker et al. reported that BP-lowering effect with HBPM was significantly intensified with co-interventions such as patients’ education, feedback, and counselling (-1.0 mmHg with HBPM alone to -6.1 mmHg with HBPM combined with intervention) [69]. The randomized trial on the efficacy of self-monitored blood pressure, with or without telemonitoring, for the titration of antihypertensive medication (TASMINH4) demonstrated that HBPM-based titration of antihypertensive medications in patients with poorly controlled BP led to a significantly greater reduction in BP than that of OBP-based titration, regardless of telemonitoring [70]. Ultimately, this approach increased the rate of BP control [13] and drug adherence, [71] and reduced medical costs [72]. Therefore, HBPM is preferred over ABPM for long-term management of hypertension. However, Staessen et al. reported that adjusting antihypertensive treatment based on ABPM or HBPM instead of OBP led to less intensive drug treatment [73]. Consequently, further studies are needed to determine whether ABPM- or HBPM-based approaches lead to a reduction in cardiovascular outcomes compared to the OBP-based approach.

#### Decision making during discrepancy between ABPM and HBPM

Ntineri et al. reported a substantial disagreement between APBM and HBPM in the diagnosis of hypertensive phenotypes (20.1%) [74]. The significant determinants of this disagreement included age, sex, study center, body mass index, cardiovascular disease history, office hypertension, antihypertensive medication, and alcohol consumption [74]. In contrast, Kim et al. failed to demonstrate any demographic or clinical features that could predict disagreements [75]. Furthermore, they suggested that individuals with a diagnostic disagreement may have an intermediate cardiovascular risk between those with sustained normotension and those with hypertension. Consequently, this diagnostic disagreement could pose challenges in clinical decision-making. However, there is no consensus regarding the therapeutic strategies when diagnostic discrepancy occurs between ABPM and HBPM. Considering the advantages and limitations of both the methods, it is crucial to take the following factors into account when discrepancies occur in clinical situations. First, physicians should evaluate HBPM techniques, including the use of non-validated device, inadequate resting time before measurements, incorrect patient positioning, improper cuff size, improper cuff positioning (not mid-arm at heart level), and alcohol consumption before measurements. In such cases, physicians should re-educate individuals on proper HBPM techniques and reassess their home BP data. Secondly, it is essential to assess proper cuff positioning and ensure that patients remain still with a relaxed arm during each ABPM measurement. Repeated ABPM should be considered in these cases. Third, physicians should assess patient discomfort such as sleep disturbances and anxiety during ABPM. In such cases, ABPM data may be inaccurate, and repeated long-term HBPM may be used instead of repeated ABPM for the management of hypertension. Fourth, physicians should recognize the inherent limitations of HBPM, including its inability to detect nocturnal hypertension. Thus, if MH or MUCH is suspected due to nocturnal hypertension, ABPM is preferred over HBPM for diagnosis and decision-making regarding hypertension. Therefore, when discrepancies occur between ABPM and HBPM, physicians should evaluate and re-educate individuals on BP measurement techniques. Furthermore, long-term follow-up with HBPM or repeated ABPM should be considered.

#### Corresponding BP

There are well-known discrepancies between OBP and out-of-office BP owing to differences in measurement techniques, time window, white coat effect, and environmental factors [76]. Therefore, correctly categorizing individual BP status requires measuring both OBP and out-of-office BP. The prevalence of BP phenotypes can vary with different OBP and out-of-office BP thresholds [77]. Consequently, determining the threshold of out-of-office BP corresponding to OBP values is crucial for the accurate diagnoses and management of hypertension. Corresponding BP levels refer to out-of-office BP levels at which the risk of cardiovascular outcomes is similar to the risk associated with corresponding OBP [76]. Cohort-based or randomized controlled trial-based studies have suggested outcome-driven corresponding ambulatory and home BP values [76]. Generally, the difference between OBP and out-of-office BP thresholds is 5 mmHg. However, different guidelines and outcome-based studies have suggested different BP levels [76, 78–80]. The Korean Society of Hypertension (KSH) recommends the same corresponding BP values for diagnosing and treating hypertension [3] (Table 1). The corresponding value of OBP 140/90 mmHg is 135/85 mmHg in both daytime ambulatory BP and home BP. However, for an OBP ≤ 130 mmHg, the corresponding out-of-office BP is comparable to OBP because the white coat effect is likely to decrease at this level (Table 1).

**Table 1:** Threshold of hypertension and corresponding BP

**1) The threshold for diagnosing hypertension**				
Category		SBP (mmHg)	DBP (mmHg)	
Office blood pressure		≥ 140	≥ 90	
Ambulatory blood pressure				
24hour		≥ 130	≥ 80	
Day		≥ 135	≥ 85	
Night		≥ 120	≥ 70	
Morning		≥ 135	≥ 85	
Home blood pressure		≥ 135	≥ 85	
**2) Corresponding BP**				
	**Office BP**	**Ambulatory BP** **(24hour)**	**Ambulatory BP** **(daytime)**	**Home BP**
Systolic BP (mmHg)	140	130	135	135
Systolic BP (mmHg)	130	125	130	130

SBP, systolic blood pressure; BP, blood pressure

## Conclusion

This review describes device selection, implementation, and clinical implication of ABPM and HBPM for accurate diagnosis and treatment of hypertension in clinical practice. To prevent device errors, validated automated oscillometric devices should be used. ABPM is performed using standard techniques and patient education is essential for proper HBPM. ABPM offers short-term comprehensive information on BP values, making it the preferred method for initial diagnosis of hypertension and for understanding BP phenotypes and circadian BP patterns. However, HBPM is easy to use and provides multiple BP readings over a long period of time, making it the preferred choice for long-term monitoring to assess the effectiveness of antihypertensive treatment. If office BP is elevated, ABPM or HBPM should be considered before initiating or up-titrating antihypertensive medications, to rule out WCH or WUCH. Additionally, even with normal or high-normal office BP, ABPM or HBPM may be considered to exclude MH or MUCH in patients with HMOD. In cases of RH, ABPM is a critical step to confirm true RH, while HBPM is useful for long-term RH management. Thus, HBPM complements the diagnosis of MH/MUCH or true RH, although it has limitations in measuring nocturnal BP. WCH in pregnant women is associated with worse perinatal and maternal outcomes than in normotensive women. Thus, if OBP is elevated in pregnant women, ABPM or HBPM is required to confirm WCH or WUCH, regardless of gestational age. Therefore, continuous administration of HBPM throughout pregnancy is advisable. When the substantial discrepancies occur between ABPM and HBPM in clinical practice, physicians should evaluate and re-educate individuals on BP measurement techniques. Furthermore, long-term follow-up with HBPM or repeated ABPM should be considered. The corresponding value of OBP 140/90 mmHg is 135/85 mmHg in both daytime ambulatory BP and home BP. However, for an OBP ≤ 130 mmHg, the corresponding out-of-office BP is comparable to OBP because the white coat effect is likely to decrease at this level.

In conclusion, out-of-office BP monitoring, including ABPM and HBPM, is essential to prevent the misdiagnosis and mistreatment of hypertension. However, it is crucial to understand that these two methods provide somewhat different information about an individual’s BP status, making it necessary to appreciate the advantages and limitations of ABPM and HBPM for the accurate diagnosis and treatment of hypertension.

## Data Availability

Not applicable.

## Abbreviations

BP: blood pressure
OBP: office blood pressure
ABPM: ambulatory blood pressure monitoring
HBPM: home blood pressure monitoring
ESH: European Society of Hypertension
WCH: white coat hypertension
MH: masked hypertension
Kor-ABP registry: Korea Ambulatory BP registry
WUCH: white coat uncontrolled hypertension
MUCH: masked uncontrolled hypertension
HMOD: hypertension-mediated organ damage
TIME: Treatment in Morning versus Evening
RH: resistant hypertension
TASMIN4: Efficacy of self-monitored blood pressure, with or without telemonitoring for the titration of antihypertensive medication
KSH: Korean Society of Hypertension
